# Six Out of Ten Women with Recurrent Urinary Tract Infections Complain of Distressful Sexual Dysfunction – A Case-Control Study

**DOI:** 10.1038/srep44380

**Published:** 2017-03-15

**Authors:** Luca Boeri, Paolo Capogrosso, Eugenio Ventimiglia, Roberta Scano, Alessandra Graziottin, Federico Dehò, Emanuele Montanari, Francesco Montorsi, Andrea Salonia

**Affiliations:** 1 Division of Experimental Oncology/Unit of Urology; URI; IRCCS Ospedale San Raffaele, Milan, 20132, Italy; 2 Department of Urology, IRCCS Fondazione Ca’ Granda – Ospedale Maggiore Policlinico; Milan, 20122, Italy; 3 University Vita-Salute San Raffaele, Milan, 20132, Italy; 4 Ospedale San Raffaele Resnati, Milan, 20132, Italy

## Abstract

Uncomplicated recurrent urinary tract infections (rUTIs) are common among reproductive-aged women. We aimed to assess the prevalence and predictors of sexual dysfunction (FSD) in a cohort of women with rUTIs and compare their psychometric scores to those of matched controls. Data from 147 rUTIs women and 150 healthy controls were analysed. Participants completed the International Prostatic Symptoms Score (IPSS), the Female Sexual Function Index (FSFI) and the Female Sexual Distress Scale (SDS). Descriptive statistics and logistic regression models tested prevalence and predictors of distressful FSD. Women with rUTIs had lower FSFI scores (p < 0.001) and a greater proportion of pathological FSFI (78.9% vs. 21.4%; p < 0.001) and SDS scores (77.8% vs. 21.4%; p < 0.001) than controls. Of rUTIs patients, 88 (60%), 77 (52.2%), and 75 (51.1%) reported pathological scores for FSFI-pain, lubrication and arousal, respectively; moreover, 64% had concomitant pathological FSFI and SDS scores. Age, IPSS severity, rUTIs, a history of ≥6 UTIs/year and a history of constipation were independent predictors of pathologic FSFI and SDS (all p ≤ 0.05). In conclusion, up to 80% of women with rUTIs showed pathologic FSFI and SDS scores, with 60% reporting scores suggestive of distressful FSD. Having ≥6 UTIs/year and a history of constipation independently predicted distressful FSD.

Uncomplicated urinary tract infections (UTIs) are among the most common bacterial infections encountered by women. Approximately 50% to 60% of women develop a UTI over their lifetime^1^, with one-third experiencing at least one by age 26^2^. A recurrent UTI (rUTI) is widely defined as more than two episodes of uncomplicated UTI within 6 months or more than three within 12 months, as documented by urine culture^3^,^4^. It has been reported that nearly 20–30% of women with an episode of UTI will have a further episode, and around 25% of these will develop rUTIs^2^. Uropathogenic *Escherichia coli* (UPEC) is the pathogen responsible 70–95% of both sporadic and rUTIs^5^.

Despite being considered a mild and self-limiting condition, rUTIs have a detrimental impact on a patient’s quality of life (QoL)^6^,^7^, producing disabling effects on women’s health, intimate and social relationships, self-esteem, and capacity for work^6^, as well as irritability, tiredness and an inability to concentrate^7^. Increasing evidence shows that sexual and urinary problems are often comorbid and possibly synergic with UTIs in women^8^,^9^,^10^. Moreover, 60% of rUTIs in women are postcoital^5^. Despite being clinically meaningful that urinary symptoms may be associated with sexual dysfunction and sexual bother, to the best of our knowledge no clinical study has examined the impact of rUTIs on women’s sexual functioning using validated psychometric questionnaires.

Given the significant prevalence of female sexual dysfunction (FSD) in the context of urologic disorders^8^,^9^,^10^ and clinical observations of sexual complaints from women with rUTIs, we cross-sectionally sought to determine the prevalence and predictors of FSD in a cohort of white–European, heterosexual, sexually active women seeking medical help for rUTI as their primary complaint, and we compared their psychometric scores with those from a group of race- and age-matched women without rUTIs.

## Results

Table 1 lists the characteristics and descriptive statistics of the entire cohort of individuals. Overall, patients with rUTIs had experienced a mean (median) of 5.29 (6) UTI episodes throughout the previous 12 months. Out of 147 women with rUTIs, 79 (53.7%) reported postcoital rUTIs. Almost 86.5% suffered from UPEC-related rUTIs, with other uropathogens (*Enterococcus faecalis, Ureaplasma urealyticum, Klebsiella, Proteus Mirabilis*) equally distributed (data not shown). Patients with rUTIs did not differ from controls in terms of age, BMI and CCI scores. Conversely, women with rUTIs more frequently showed a history of constipation (p = 0.006), were more frequently current smokers (p = 0.02) and were less likely to be in a stable sexual relationship (p = 0.04).

Table 2 depicts the descriptive statistics of the psychometric scores assessing sexual functioning and sexual distress among the cohort. Women with rUTIs showed significantly lower mean FSFI scores (p < 0.001) and had pathological FSFI scores more often than controls (78.9% vs. 21.4%; p < 0.001). When stratified, all FSFI sub-domains were significantly lower in the rUTIs than in –rUTIs individuals, with the exception of sexual desire. In the rUTIs group, 88 (60%), 77 (52.2%) and 75 (51.1%) women reported pathological FSFI-pain, lubrication and arousal subdomains, respectively. Similarly, patients with rUTIs had higher mean SDS scores (p < 0.001), and more pathological SDS scores (77.8% vs. 21.4%; p < 0.001) than controls. Overall, 64% of rUTIs women had concomitant pathological FSFI and SDS scores.

Considering only the rUTIs group, mean FSFI scores decreased with age (p = 0.03) and with increasing IPSS severity (p = 0.002) (Table 3). Moreover, the FSFI score was significantly lower in women with a history of constipation (p < 0.001). Women with ≥6 episodes of UTI over the last 12 months reported worse FSFI, SDS and IPSS scores (all p ≤ 0.04), respectively, compared to women with <6 UTIs per year. FSFI, SDS and IPSS scores did not vary according to BMI or CCI categories (Table 3).

Table 4 reports psychometric scores assessing urinary symptoms according to rUTIs status. Women with rUTIs showed higher mean IPSS scores (p = 0.003) and more frequently reported pathological storage symptoms than controls (p = 0.01). Conversely, no difference was observed between groups for voiding symptoms. Similarly, the IPSS-QoL domain score was higher in patients with rUTIs (p < 0.001).

Table 5 details the logistic regression models testing the associations between clinical predictors and pathologic psychometric scores. Model A, considering all participants, showed that age, total IPSS score and rUTIs were each associated with pathologic FSFI scores (all p ≤ 0.01). Conversely, CCI ≥1 and BMI were not. Similar findings were found for SDS scores. At logistic MVA, age, total IPSS score and rUTIs achieved independent predictor status for pathologic FSFI scores (all p ≤ 0.04) and for pathologic SDS scores (all p ≤ 0.02). Model B, focusing only on women with rUTIs, showed that age, total IPSS score, having ≥6 UTI episodes in the previous 12 months and a history of constipation were univariably associated with pathologic FSFI scores (all p ≤ 0.01). Similar findings were found for SDS scores. At logistic MVA, age, total IPSS score, having ≥6 UTIs in the previous 12 months, and a history of constipation achieved independent predictor status for both pathologic FSFI scores (all p ≤ 0.02) and pathologic SDS scores (all p ≤ 0.04).

## Discussion

This study used validated psychometric tools to compare FSD prevalence and predictors in women with rUTIs to a group of same-age women not reporting UTI. Of clinical importance, nearly 8 out of 10 women with rUTIs reported scores suggestive for FSD. Likewise, women with rUTIs more frequently reported FSD compared to controls, with a significant impairment on the FSFI-pain, lubrication and arousal domains. Age, severity of LUTS, a history of ≥6 episodes of UTI over the previous 12 months and a history of constipation were independent predictors of FSD among rUTIs patients.

Our interest was fuelled by increasing evidence regarding the relationship between LUTS and FSD^8^,^11^. Several studies have suggested that women with OAB have high rates of sexual impairment^11^,^12^, and lower mean scores for all domains of the FSFI, compared to healthy individuals^13^. Similarly, urgency and urinary incontinence (UI) have been frequently associated with FSD^14^,^15^.

The current results confirm previous findings showing a connection between LUTS and FSD. In fact, the higher the IPSS score in patients with rUTIs, the greater the progressive impairment of both FSFI and SDS scores. Previous studies assessing the relationship between LUTS and FSD found that 60% of patients with sexual arousal disorders and 61% with sexual pain disorders also complained of rUTIs^8^,^13^. Although the potential detrimental impact of rUTIs on QoL has been previously suggested^6^,^7^, the actual impact on women’s sexual functioning had never been comprehensively analysed. Therefore, the current findings are clinically relevant, because i) rUTIs are quite frequent in daily clinical practice; ii) they provide novel evidence for an association between rUTIs and FSD in sexually active, healthy women; iii) rUTIS were not only post-coital, as in previously published series^16^.

Taken together, the results of this real-life case-control study show that FSD prevalence among women with rUTIs was actually greater than that reported among women with classical LUTS^8^,^11^,^13^. Further corroborating this finding, sexual dysfunction was found in 47% of women with OAB and LUTS/UI^8^,^13^ while some degree of sexual impairment was reported by 19% to 50% of women with UI or pelvic floor disorders^11^,^17^. Focusing on the FSFI sub-domains, we found that women with rUTIs reported pathological scores of all domains, with the exception of sexual desire. These findings are in agreement with the complexity of sexual dysfunction previously observed among individuals suffering from LUTS/UI^8^, pure OAB^13^ or UI^18^ as a primary compliant.

Importantly, the FSFI-pain score was not only lower in patients with rUTIs compared to healthy women, but was pathological. To this regard, Salonia *et al*. recently showed that three out of five sexually active women of reproductive age whose primary complaint was rUTIs also suffered from secondary provoked vestibulodynia^10^.

We found that FSFI and SDS impairments were associated with age at presentation. These results corroborate previous findings showing that younger women tend to have greater sexual distress associated with urinary problems^13^,^19^, while women experiencing the menopause transition frequently experience a significant reduction of oestrogens at the genital level, which may be associated with or enhanced by the rUTIs themselves^20^. Importantly, to avoid confounding the effects of rUTI with effects experienced by post-menopausal women, in whom specific syndromes such as vulvovaginal atrophy become chronic and progressive^21^,^22^, we did not include post-menopausal women in the current study.

Recurrent cystitis is sometimes implicated with inflammation of the genitalia, vaginal lubrication disturbance and higher incidence of pain disorders. Moreover, vaginal dryness and low lubrication caused by urine in the vagina affects the normally acidic pH and may play a role in patients’ complaints. One could speculate that rUTIs may eventually trigger pelvic floor overactivity, thus predisposing the area to both microabrasion of the vestibular mucosa and microtrauma of the urethral area contributing to pain and discomfort during sexual intercourse. Moreover, rUTIs may lead to inflammation of the bladder mucosa with hyperactivation of the mast cells in the bladder wall and subsequent proliferation of pain nerve fibres in the bladder and urethral wall, leading to hyperalgesia and allodynia during intercourse^16^.

Several data showed that cigarette smoking is one of the greatest risk factors for the development of bladder cancer^23^; likewise, the impact of smoking on promoting inflammation per se has been extensively reported. However, the impact of cigarette smoking on inducing bladder inflammation has been scantly analysed. Only one preclinical study illustrated the potential link between cigarette smoking and bladder inflammation mediated by the platelet-activating factor (PAF) accumulation and subsequent recruitment of inflammatory cells (i.e. mast cells) in the bladder wall^24^. Of clinical relevance, we found that women with rUTIs were more frequently current smokers that those without rUTIs. Thereof, it is possible to speculate that an increase in bladder inflammation mediated by cigarette smoking could act as an additional contributing factor for the development of rUTIs.

Of note, patients with rUTIs were less likely to be in a stable sexual relationship than controls in our cohort. This result is consistent with previous findings in premenopausal women where having multiple partners was reported as a strong behavioural risk factor for rUTIs^1^. Indeed, in a case-control study of women aged 18–30 years, Scholes *et al*.^25^ showed that those with rUTIs had more frequent sexual intercourse and reported higher numbers of sexual partners than those without rUTIs. Overall, these risk factors were found to increase vaginal and urethral colonization with *E. Coli*^1^.

Even of clinical relevance, we found that women with rUTIs and a history of constipation had a 6-fold and 5-fold higher risk of having pathologic FSFI and SDS scores, respectively. The significant association between constipation history and FSD is corroborated by the close relationship between constipation itself and the incidence of rUTIs^26^. Some studies have suggested that the foecal flora acts as an optimal reservoir for the microbial strain that may subsequently recolonize the introitus and then the bladder, causing rUTIs^26^,^27^.

A potential strength of the study is that patients were enrolled at an outpatient clinic they had visited with a primary complaint of UTIs, therefore representing a typical real-life scenario. Additionally, this study highlights the importance of caregivers being more aware of the impact of rUTIs on women’s sexuality. Unfortunately, despite the scientific community frequently reporting the association between FSD and LUTS in women, this issue is still largely unknown or misunderstood by urologists and gynaecologists^28^. Thus, we are only beginning to understand the causal/epiphenomenal relationship between rUTIs and distressful FSD. Therefore, a dialogue about sexual function in women with urinary infections should become an integral component of clinical management.

Our study is not devoid of limitations. The results were derived from a relatively small, homogenous cohort of women and deserve external validation with an independent, larger and more diverse sample. Our analyses lacked precise data on the frequency of penetrative vaginal or anal intercourse. Moreover, we also lacked comprehensive information regarding the specific real-time culture and thus were unable to exclude the possibility of other vaginal pathogens not found in the urine samples. Further, we lacked data regarding the local hormonal milieu, which is clearly important for women’s urogynecologic and sexual health. Finally, being a real-life study we lack a baseline FSFI assessment before patients developed rUTIs.

In conclusion, this case-control study reports novel findings regarding the high prevalence of distressful sexual dysfunction among women suffering from rUTIs: nearly 80% of sexually-active women affected by rUTIs had psychometric scores suggestive of sexual dysfunction. Overall, distressful sexual dysfunction emerged as a complex entity involving more than one aspect of sexual functioning, with the most significant impairments concerning sexual pain, lubrication and arousal. Of clinical importance, we found a positive association between FSD and patient age, severity of LUTS and a history of bowel constipation. Age, LUTS severity, having more than 6 episodes of UTIs throughout the previous 12 months, and a history of constipation were independent predictors of FSD. Overall, the current results indicate a clinical need for comprehensive investigations into the sexual health of women with UTIs, keeping in mind the extraordinary epidemiological impact that these uncomplicated infections can have on a woman’s quality of life.

## Methods

From January 2014 to January 2016, demographic and clinical variables from 147 consecutively treated, white–European, heterosexual, sexually-active women seeking medical help for symptomatic rUTIs at the same urologic outpatient clinic were considered for this exploratory, prospective analysis. An additional 3 patients were enrolled in the study but ultimately excluded due to incomplete data collection.

Patients were assessed with a thorough medical and sexual history. Health-significant comorbidities were scored with the Charlson Comorbidity Index (CCI)^29^, using the International Classification of Diseases, 9^th^ revision. For our analysis, CCI was categorised as 0 or ≥1. All individuals were sexually active, with at least four occasions of sexual intercourse per month. The body mass index (BMI) of each patient was classified using the cut offs proposed by the National Institutes of Health (NIH): normal weight (18.5–24.9), overweight (25.0–29.9), and class ≥1 obesity (≥30.0).

Patients were excluded if younger than 18 or older than 50, if they had used combined hormonal contraception during the previous 6 months; if they were postmenopausal (either natural or surgical/iatrogenic), pregnant, breastfeeding or trying to conceive, if they had symptoms of upper UTI, a history of urinary tract anomalies/vesicoureteral reflux, interstitial cystitis, diabetes, urinary tract stones, urinary incontinence, neurologic conditions, clinical depression or depressive symptoms. A pelvic ultrasound assessment was performed in every patient during the baseline workup to rule out potentially relevant post-void residual. None of the patients in the study group had an indwelling catheter or used self-catheterization for any bladder dysfunction.

Urine cultures obtained through clean-catch samples (void after cleaning) were analysed. The microbiologic definition of UTI was 10^5^ or more colony-forming units per milliliter from midstream. Recurrent UTI is defined as more than two symptomatic episodes of uncomplicated UTI within 6 months, or more than three within 12 months, as documented by culture^3^,^4^. Symptomatic UTIs were defined through a detailed medical history.

Patients were defined as being in a stable relationship if they had the same sexual partner for 12 or more consecutive months. Prevalence of lower urinary tract symptoms (LUTS) was analysed in all subjects.

A total of 150 healthy age-matched sexually active women assessed in a routine gynaecological evaluation and no complaints of rUTIs (-rUTIs) over the last 3 years at least, were used as a cross-sectional control group.

All participants underwent a standard pelvic exam and no indications of abnormality were observed in any of the women in either group.

To provide a frame of reference for interpreting urinary dysfunction, all participants completed the International Prostatic Score Symptoms (IPSS)^30^.

To assess sexual dysfunction and sexual distress profiles, patients also completed The Female Sexual Function Index (FSFI)^31^ and the Female Sexual Distress Scale (SDS)^32^. The FSFI was categorized according to the cut-off value of 26.55 proposed by Wiegel *et al*., whereas SDS was dichotomized according to the median value^33^.

Data collection was carried out following the principles outlined in the Declaration of Helsinki; after approval of the IRCCS San Raffaele Hospital’s Ethical Committee, all patients signed an informed consent agreeing to supply their own anonymous data for this and future studies.

Data are presented as means (SD; ranges). The statistical significance of differences in means and proportions was tested with the one-way analysis of variance (ANOVA) and Pearson chi-square test, respectively. A 95% confidence interval was estimated for the association of categorical parameters. Exploratory analyses were initially applied to all variables; variables were retained for analysis when deemed clinically significant to the results. We performed two logistic regression models. Model A assessed potential predictors of FSD in the whole population. Logistic regression univariable analysis (UVA) and multivariable analysis (MVA) tested the associations between clinical predictors (e.g. age, BMI, CCI, IPSS, rUTIs) and pathologic psychometric scores (FSFI and SDS). Conversely, model B tested the association between predictors (age, BMI, CCI, IPSS, number of UTI episodes in the last 12 months according to median value, constipation history) and the presence of pathologic psychometric scores in women with rUTIs. Statistical analyses were performed using SPSS statistical software, version 20 (IBM Corp, Armonk, NY). All tests were 2-sided with a significance level of 0.05.

## Additional Information

**How to cite this article:** Boeri, L. *et al*. Six Out of Ten Women with Recurrent Urinary Tract Infections Complain of Distressful Sexual Dysfunction – A Case-Control Study. *Sci. Rep.* **7**, 44380; doi: 10.1038/srep44380 (2017).

**Publisher's note:** Springer Nature remains neutral with regard to jurisdictional claims in published maps and institutional affiliations.

## Figures and Tables

**Table 1:** Baseline characteristics and descriptive statistics of participants (No. = 297).

	−rUTIs	+rUTIs	p value (F)*
No. of patients	150 (50.5%)	147 (49.5%)	
Age (years)			
Mean (SD)	34.5 (6.0)	35.3 (9.2)	0.71 (0.13)
Range	24–48	19–50	
BMI [kg/m^2^]			0.32 (0.99)
Mean (SD)	21.3 (2.3)	22.0 (3.7)	
Range	17.6–29.4	15.6–35.4	
BMI (NIH classification) [No. (%)]			0.39 (*X*_*2*_ = 1.85)
18.5–24.9	139 (92.9)	124 (84.4)	
25–29.9	11 (7.1)	16 (10.9)	
≥30	0 (0.0)	7 (4.8)	
CCI			0.83 (0.43)
Mean (SD)	0.29 (0.6)	0.26 (0.6)	
Range	0–2	0–3	
CCI categorized [No. (%)]			0.45 (*X*_*2*_ = 0.56)
0	118 (78.6)	124 (84.4)	
≥1	32 (21.4)	23 (15.6)	
Relationship status [No. (%)]			0.04 (*X*_*2*_ = 4.01)
Stable sexual relationship	61 (41.5)	32 (21.4)	
No stable sexual relationship	86 (58.5)	118 (78.6)	
Educational Status [No. (%)]			0.25 (*X*_*2*_ = 2.77)
Primary/Secondary school	16 (10.7)	35 (23.8)	
High school	91 (60.7)	69 (46.9)	
University+	43 (28.6)	43 (29.3)	
Bowel History [No. (%)]			0.006 (*X*_*2*_ = 7.51)
Normal	91 (60.7)	49 (33.3)	
Constipation	59 (39.3)	98 (66.7)	
Current smokers [No. (%)]	79 (52.8)	94 (63.9)	0.02 (*X*_*2*_ = 7.98)
Alcohol drinkers [No. (%)]	100 (66.6)	90 (61.2)	0.46 (*X*_*2*_ = 1.55)
No. of UTI episodes (prior 12 months)			
Mean (SD)	—	5.29 (6.0)	
Median	—	6	
Range	—	3–8	

Keys: -rUTIs = Negative for recurrent urinary tract infections; +rUTIs = Positive for recurrent urinary tract infections; BMI = body mass index; CCI = Charlson Comorbidity Index.

*P value according to chi-square test or analysis of variance (ANOVA), as indicated.

**Table 2:** Psychometric scores assessing sexual function and sexual distress in the whole cohort (No. = 297).

	−rUTIs (No. = 150)	+rUTIs (No. = 147)	p-value (F)*
FSFI total score			<0.001 (69.23)
Mean (SD)	63.7 (9.7)	17.6 (9.0)	
Range	7.0–92.0	1.2–35.4	
Categorized FSFI total scores [No. (%)]			<0.001 (*X*_*2*_ = 31.1)
Normal	118 (78.6)	31 (21.1)	
Pathological	32 (21.4)	116 (78.9)	
FSFI domains, Mean (SD)			
Desire	4.0 (1.2)	3.7 (1.3)	0.21 (5.44)
Arousal	4.1 (1.9)	2.7 (1.8)	0.001 (10.7)
Lubrication	4.3 (2.1)	2.6 (1.8)	<0.001 (16.9)
Orgasm	4.3 (2.1)	2.8 (2.3)	<0.01 (7.04)
Satisfaction	4.4 (2.2)	3.4 (1.9)	0.02 (5.41)
Pain	3.9 (2.2)	2.6 (1.7)	0.001 (10.76)
Pathological FSFI domains [No. (%)]			
Desire	47 (31.2)	52 (35.6)	0.08 (*X*_*2*_ = 3.28)
Arousal	39 (25.9)	75 (51.1)	0.02 (*X*_*2*_ = 5.79)
Lubrication	32 (21.4)	77 (52.2)	0.008 (*X*_*2*_ = 7.10)
Orgasm	27 (17.9)	69 (46.7)	0.01 (*X*_*2*_ = 6.66)
Satisfaction	43 (28.6)	74 (50.1)	0.04 (*X*_*2*_ = 4.37)
Pain	48 (32.1)	88 (60.0)	0.007 (*X*_*2*_ = 7.39)
SDS.			<0.001 (31.8)
Mean (SD)	8.9 (6.9)	23.1 (12.6)	
Range	0–25	0–47	
Categorized SDS Scores [No. (%)]			<0.001 (*X*_*2*_ = 29.5)
Normal	118 (78.6)	33 (22.2)	
Pathological	32 (21.4)	114 (77.8)	
Concomitant SDS/FSFI scores [No. (%)]			
nSDS/nFSFI	91 (60.7)	11 (7.8)	<0.001 (*X*_*2*_ = 36.4)
pSDS/pFSFI	6 (3.6)	95 (64.4)	<0.001 (*X*_*2*_ = 31.6)

Keys: -rUTIs = Negative for recurrent urinary tract infections; +rUTIs = Positive for recurrent urinary tract infections; FSFI = Female Sexual Function Index; SDS = Sexual Distress Scale; nSDS/FSFI = normal SDS/FSFI; pSDS/FSFI = pathological SDS/FSFI; *P value according to chi-square test or analysis of variance (ANOVA), as indicated.

**Table 3:** Psychometric scores and clinical characteristics only in the groups of patients with rUTIs (No. = 147; [Mean (SD)].

	FSFI	p-value (F)	SDS	p-value (F)	IPSS	p-value (F)
Age		0.03 (3.5)		0.04 (3.2)		0.04 (3.2)
18–25	24.1 (12.4)		11.2 (15.4)		8 (9.9)	
26–35	21.4 (9.6)		21.4 (11.3)		10.4 (5.2)	
36–50	16.5 (8.3)		24.1 (12.2)		13.2 (6.6)	
BMI		0.87 (0.13)		0.14 (1.97)		0.27 (1.3)
18.5–24.9	17.6 (9.0)		23.4 (12.5)		12.3 (6.5)	
25–29.9	18.4 (11.1)		15.6 (13.0)		13.1 (7.5)	
≥30	15.3 (3.1)		30.3 (9.2)		18.6 (12.0)	
CCI		0.61 (0.26)		0.49 (0.47)		0.72 (0.13)
0	17.7 (8.8)		19.8 (17.3)		11.61 (4.1)	
≥1	15.9 (11.0)		23.3 (12.2)		12.66 (7.0)	
Bowel History		<0.001 (21.2)		<0.001 (24.2)		<0.001 (13.4)
Normal	25.5 (8.7)		11.3 (10.6)		7.67 (6.2)	
Constipation	15.6 (7.9)		25.9 (11.4)		13.8 (6.4)	
No. of UTI episodes (prior 12 months)		0.012 (6.7)		0.002 (10.3)		0.04 (3.51)
≥6	15.7 (9.1)		25.5 (13.5)		13.1 (6.6)	
≤5	21.8 (9.5)		15.1 (12.1)		8.9 (6.3)	
Categorized IPSS score		0.002 (5.44)		<0.001 (6.78)		
None	27.5 (2.2)		4.3 (4.5)			
Mild	21.8 (8.8)		19.43 (12.)			
Moderate	15.4 (7.5)		23.1 (11.9)			
Severe	14.8 (8.6)		31.6 (9.6)			

Keys: rUTIs = recurrent urinary tract infections; IPSS = International Prostatic Symptoms Score; BMI = body mass index.

CCI = Charlson Comorbidity Index.

*P value according to ANOVA test of variance.

**Table 4:** Psychometric scores assessing urinary symptoms in the whole cohort (No. = 297).

	−rUTIs (No. = 150)	+rUTIs (No. = 147)	p value (F)*
Total IPSS score			0.003 (9.43)
Mean (SD)	6.3 (5.1)	10.4 (6.5)	
Range	0–23	0–30	
Categorized IPSS scores [No. (%)]			0.04 (*X*_*2*_ = 8.01)
None	21 (14.3)	5 (3.3)	
Mild	75 (50.0)	56 (37.8)	
Moderate	48 (32.1)	65 (44.4)	
Severe	6 (3.6)	21 (14.4)	
IPSS – Storage Scores			<0.001 (14.1)
Mean (SD)	3.8 (2.7)	6.6 (3.7)	
Range	0–20	0–20	
Categorized Storage symptoms [No. (%)]			0.01 (*X*_*2*_ = 9.14)
None	27 (19.3)	20 (13.3)	
Mild	52 (34.6)	34 (23.3)	
Moderate to severe	71 (47.1)	93 (63.3)	
IPSS – Voiding symptoms			0.12 (2.47)
Mean (SD)	2.5 (3.1)	3.8 (3.9)	
Range	0–12	0–18	
Categorized Voiding Scores [No. (%)]			0.01 (*X*_*2*_ = 9.20)
None	59 (39.3)	20 (13.3)	
Mild	64 (42.9)	87 (58.9)	
Moderate to severe	27 (17.9)	40 (27.8)	
IPSS – QoL score			<0.001 (60.1)
Mean (SD)	0.9 (1.1)	3.4 (1.6)	
Range	0–3	0–6	

Keys: -rUTIs = Negative for recurrent urinary tract infections; + rUTIs = Positive for recurrent urinary tract infections;

IPSS = International Prostatic Symptoms Score; QoL = Quality of life.

*P value according to chi-square test or analysis of variance (ANOVA), as indicated.

**Table 5:** Logistic regression models predicting pathologic psychometric scores (OR; *p* value [95%CI]) in the whole cohort (Model A; No. = 297) and only in women with rUTIs (Model B; No. = 147).

	Pathologic FSFI		Pathologic SDS		Pathologic FSFI		Pathologic SDS	
	UVA	MVA	UVA	MVA	UVA	MVA	UVA	MVA
**Model A**					**Model B**			
Age	1.3; <0.001 [1.06–1.2]	1.10; 0.03 [1.05–1.15]	1.16; <0.002 [1.08–1.24]	1.08; 0.025 [1.01–1.17]	1.3; <0.001 [1.06–1.2]	1.22; 0.009 [1.05–1.4]	1.16; <0.002 [1.08–1.24]	1.15; 0.021 [1.02–1.3]
BMI	1.05; 0.40 [0.93–1.2]	1.06; 0.44 [0.96–1.24]	1.01; 0.76 [0.9–1.14]	1.01; 0.89 [0.86–1.18]	1.05; 0.40 [0.93–1.2]	1.05; 0.65 [0.84–1.30]	1.01; 0.76 [0.9–1.14]	0.96; 0.66 [0.79–1.15]
CCI ≥1	0.58; 0.36 [0.18–1.8]	1.72; 0.53 [0.32–9.36]	0.47; 0.53 [0.9–1.14]	0.48; 0.38 [0.09–2.42]	0.58; 0.36 [0.18–1.8]	1.15; 0.91 [0.08–8.81]	0.47; 0.53 [0.9–1.14]	0.18; 1.21 [0.02–1.55]
Tot IPSS	1.2; <0.01 [1.08–1.3]	1.10; 0.04 [1.02–1.2]	1.26; <0.001 [1.14–1.3]	1.2; 0.002 [1.06–1.30]	1.2; <0.01 [1.08–1.3]	1.21; 0.02 [1.02–1.4]	1.26; <0.001 [1.14–1.3]	1.2; 0.015 [1.04–1.41]
+rUTIs	13.7; <0.001 [4.86–8.5]	7.69; <0.001 [2.49–7.78]	12.83; <0.001 [4.58–13.9]	5.68; 0.003 [1.77–18.16]	—	—	—	—
					—	—	—	—
No. of UTI episodes (over the prior 12 months) ≥6 vs.<6					6.2; 0.003 [1.9–20.3]	9.3; 0.02 [1.32–6.2]	7.31; <0.001 [2.38–22.3]	6.8; 0.022 [1.31–5.30]
Constipation history Yes vs. No					12.3; <0.001 [3.8–20.2]	6.32; 0.03 [1.22–6.6]	16.1; <0.001 [4.8–18.3]	4.9; 0.04 [1.08–9.8]

Keys: UVA = Univariable analyses; MVA = Multivariable analyses; CCI = Charlson Comorbidity Index; BMI = body mass index; +rUTIs = Positive for recurrent urinary tract infections.
